# Proteomic and Properties Analysis of Botanical Insecticide Rhodojaponin III-Induced Response of the Diamondback Moth, *Plutella xyllostella* (L.)

**DOI:** 10.1371/journal.pone.0067723

**Published:** 2013-07-05

**Authors:** Xiaolin Dong, Yifan Zhai, Meiying Hu, Guohua Zhong, Wanjun Huang, Zhihua Zheng, Pengfei Han

**Affiliations:** 1 Laboratory of Insect Toxicology, College of Resources and Environment, South China Agricultural University, Guangzhou, China; 2 School of Life Sciences, Sun Yat-sen University, Guangzhou, Guangdong, China; University of South Florida College of Medicine, United States of America

## Abstract

**Background:**

Rhodojaponin III, as a botanical insecticide, affects a wide variety of biological processes in insects, including reduction of feeding, suspension of development, and oviposition deterring of adults in a dose-dependent manner. However, the mode of these actions remains obscure.

**Principal Findings:**

In this study, a comparative proteomic approach was adopted to examine the effect of rhodojaponin III on the *Plutella xyllostella* (L.). Following treating 48 hours, newly emergence moths were collected and protein samples were prepared. The proteins were separated by 2-DE, and total 31 proteins were significantly affected by rhodojaponin III compared to the control identified by MALDI-TOF/TOF-MS/MS. These differentially expressed proteins act in the nervous transduction, odorant degradation and metabolic change pathways. Further, gene expression patterns in treated and untreated moths were confirmed by qRT-PCR and western blot analysis. RNAi of the chemosensory protein (PxCSP) gene resulted in oviposition significantly increased on cabbage plants treated with rhodojaponin III.

**Conclusions:**

These rhodojaponin III-induced proteins and gene properties analysis would be essential for a better understanding of the potential molecular mechanism of the response to rhodojaponin III from moths of *P. xylostella*.

## Introduction

Insects can recognize a variety of plant compounds, which stimulate specific behaviors, such as feeding and egg laying (oviposition) by chemoreceptive organs [1], [2]. It is well known that some insects lay eggs on their host plants, and the oviposition behavior is induced by the recognition of the plant compounds with sensilla on these chemoreceptive organs [3], [4]. There are many binding proteins on these sensilla, such as general odorant-binding proteins (GOBPs), pheromone binding proteins (PBPs) and chemosensory proteins (CSPs) as well as potent odorant-degrading enzymes (ODEs) [5]–[7]. The perceptions from environment require rapid enzymatic degradation of the active chemical signal in the sensory hairs and would allow the neurons to respond chiefly to new incoming signal and enable the moth to quickly detect external fluctuations in pheromone concentration and adjust its flight behavior accordingly [8], [9]. However, the molecule mechanism of this identification and degradation is not clearly understood.

The widespread use of synthetic, broad-spectrum insecticides has being concerned because the possible hazardous effects on the environment and human health, resistance development in insect populations, and so forth [10]. Considerable efforts are being made worldwide to find safer, biodegradable substitutes for these synthetic insecticides. Research in recent years has been turning more towards selective biorational pesticides. Several botanical species were used as sources of insecticides [11]. *Rhododendron molle* (B.) G. Don (Ericaceae) has long been used for insecticidal and medicinal purposes, and rhodojaponin III was verified as the main potent component of the biologically active compound [12], [13]. Unlike some volatile chemicals [5], rhodojaponin III is a nonvolatile plant secondary metabolite like strophanthidin glycoside [14], [15]. Previous reports demonstrated that rhodojaponin III had an intense oviposition-deterring activity against many insects [15]. Although chemical studies of these deterrents in plants have been reported for several species of insect pests, the mechanism of the chemoreception and response profilings in insect is not clear.

In order to avoid multitude aggressions from external sources, animals have developed a wide variety of defensive mechanisms. At the very simplest these mechanisms include moving away from sources of attack, whilst the most complex are those of the multifaceted physiology and biochemistry variation [16]. To prevent the accumulation of residual stimulant and hence sensory adaptation, the external molecules are subsequently inactivated by much slower enzymatic degradation [17]. But the biochemistry mechanisms involved in this sequence of events is largely unknown.

The diamondback moth, *Plutella xylostella* (L.), has become the most destructive insect of cruciferous plants throughout the world, especially on the vegetables and oil crops. The damage caused by this insect results significant losses and the global spend US $1.0 billion on controlling it annually [18], [19]. For its character of high fecundity, overlapping generations and genetic plasticity, especially the various insecticides selection pressures, it has developed resistance to many synthetic insecticides [20], [21]. So there is an urgent need to develop new pest control strategies against the *P. xylostella*. The deeper understanding of the molecular mechanisms of its oviposition behaviors is essential for developing effective approaches to solve this problem. Reproduction is the basis for the proliferation of a pest population. Of special importance to pest oviposition is to find the appropriate position.

Although gene sequencing has been greatly promoted by progress in structural and functional genomics, the functions of proteins that depend on post-translational and protein-protein interactions cannot be induced only through genomic analysis. Proteomics has presented as a powerful method to gain insight into physiological changes at the protein level [22], [23]. Two-dimensional gel electrophoresis (2-DE) combined with mass spectrometry (MS) has been frequently used in insect proteomics research [24], [25]. The identification of differential proteins is a prerequisite for following and understanding the biochemical mechanism by which rhodojaponin III exerts its effects. In addition, the present results may obtain a better understanding of molecular mechanism about rhodojaponin III-induced oviposition-deterring and insect response to it.

## Results

### 2-DE Analysis of Differentially Expressed Proteins

**Figure 1:**
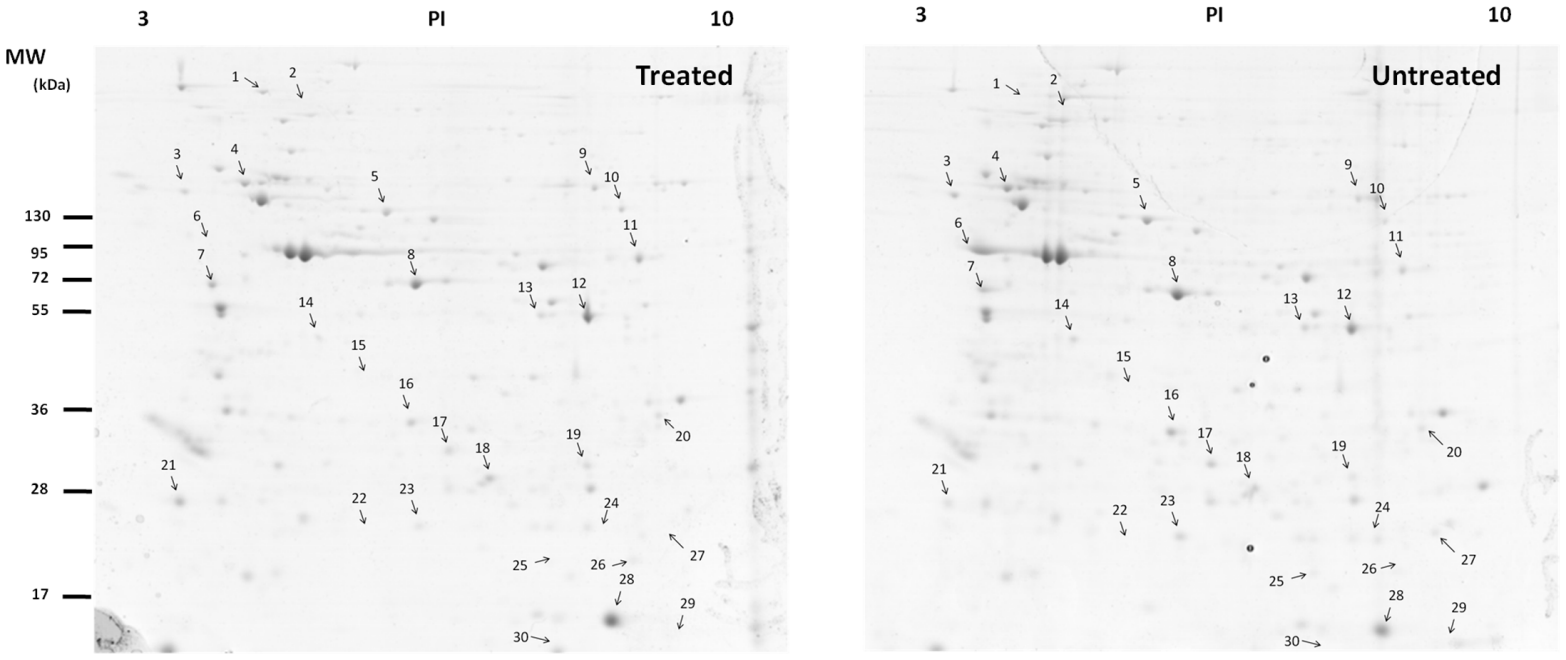
Two-dimensional electrophoresis map of proteins in *p. xylostella*. untreated (control), treated (exposed to rhodojaponin III).

**Figure 2:**
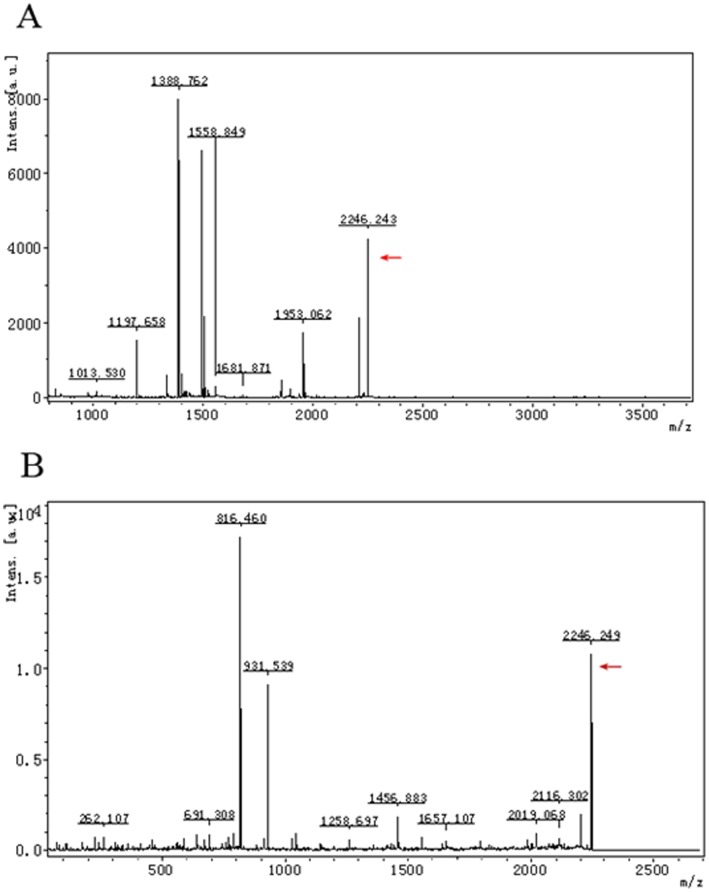
Representative PMF and MS/MS spectra. Spot 16 is identified as ArgK. (A) PMF spectrum; (B) MS/MS spectrum.

To investigate the differentially expressed proteins between control and 0.5 g/L rhodojaponin III exposed moths, we carried out a two-dimensional gel electrophoresis (2-DE) analysis (Fig. 1), and the spot 16 was detected Arginine kinase-like protein (ArgK) (Fig. 2). Each sample was subjected to triplicate runs, and the results were highly reproducible. The images were analyzed using the PD-Quest analysis software. Approximately 350 protein spots were detected on the gel. The majority of spots distributed on the map had pI values ranging from 4.0 to 9.0 and molecular weight from 10 to 200 kDa. After matching analysis, 31 protein spots were significantly different between these two gels.

### Identification of the Differentially Expressed Proteins by LC-MS/MS

Differentially expressed protein spots between control and 0.5 g/L rhodojaponin III exposed moths were subsequently were subjected to in-gel digestion and MALDI-TOF/TOF-MS/MS analysis. 31 differentially expressed proteins were identified in two different groups. Among these identified proteins, 10 proteins were up-regulated and 21 proteins were down-regulated (Table 1).

**Table 1:** Identification of Differentially Expressed protein spots.

Spot no.^a^	Accession no.	Protein description	theoretical(p*I*/kDa)	database	Matched peptides^b^	Unmatched peptides^c^	Coverage (%)	E- values	Protein scroe^d^	Peptides identified^e^
**20**	gi|195963353	3-hydroxyisobutyrate dehydrogenase *[Bombyx mori]*	9.14/34.02	Inv_others *Px*_EST	1	6	4	5.6e-08	66	R.SPIPLGAVATQLYR.I
**21**	gi|237636932	14-3-3 zeta *[Heliothis virescens]*	4.84/28.1	NCBInr	8	24	36	4.4e-43	472	R.NTVVEDSQKAYQDAFEISK.S
**12**	gi|357618425	putative cxpwmw03 *[Danaus plexippus]*	7.71/34.74	NCBInr	4	13	8	3.5e-14	168	R.AAVDAGFVPNDLQIGQTGK.I
**15**	gi|270298186	Enolase *[Pieris rapae]*	5.58/47.12	Inv_others *Px*_EST	12	23	62	3.5e-65	583	K.FGLDSTAVGDEGGFAPNIQNNK.E
**26**	gi|3582502	glutathione S-transferase isozyme 3 *[Plutella xylostella]*	8.5/24.1	*Px*_EST	2	7	6	1.1e-15	176	R.AVTFLIFTEGLK.K
**17**	gi|85165	tropomyosin, exon 9B - fruit fly *[Drosophila melanogaster]*	4.67/32.9	NCBInr	3	8	5	8.8e-13	159	K.ALQNAESEVAALNRR.I
**16**	gi|284927832	arginine kinase-like protein *[Plutella xylostella]*	5.76/39.66	Inv_others *Px*_EST	4	16	6	1.4e-38	378	R.LGFLTFCPTNLGTTVR.A
**28**	gi|301508512	apolipophorin-III *[Plutella xylostella]*	7.91/18.4	*Px*_EST	4	12	8	3.5e-23	262	R.EAPAGSTQLQDLEK.H
**6**	gi|53148459	tropomyosin I *[Plutella xylostella]*	4.74/32.54	NCBInr	1	14	3	7e-08	79	K.LLEAQQSADENNR.M
**13**	gi|357614862	acyl-CoA dehydrogenase *[Danaus plexippus]*	7.51/48.5	NCBInr	4	4	6	2.2e-25	261	K.IYQIYEGTSQIQR.L
**18**	gi|22450121	glyceraldehyde-3-phosphate dehydrogenase *[Plutella xylostella]*	6.54/35.47	*Px*_EST	5	11	18	4.4e-27	395	K.LISWYDNEYGYSNR.V
**19**	gi|357607952	fructose 1,6-bisphosphate aldolase *[Danaus plexippus]*	8.09/42.03	NCBInr	2	12	6	5.6e-09	115	R.IVPIVEPEVLPDGEHDLDR.A
**23**	gi|300470333	glutathione S-transferase delta *[Plutella xylostella]*	6.32/23.92	*Px*_EST	12	7	36	7e-66	524	R.FGDYFYPQLFGGAPEDKEK.L
**5**	gi|112982822	phosphoglyceromutase *[Bombyx mori]*	6.33/28.6	NCBInr	2	8	5	4.5e-10	125	K.AEGYQFDVAHTSVLKR.A
**22**	gi|49532926	Glutathione S transferase 2-like protein *[Plutella xylostella]*	5.85/23.53	*Px*_EST	4	7	12	4.6e-25	295	R.RPDLDQQYPGFAK.V
**8**	gi|328670887	voltage-dependent anion-selective channel *[Helicoverpa armigera]*	6.96/30.1	NCBInr	2	7	8	3.5e-15	197	K.YAVKDYGLTFTEK.W
**31**	gi|209978476	Chemosensory protein *[Plutella xylostella]*	6.88/15.45	NCBInr	2	16	4	5.6e-12	135	K.CVLDQGKCSPDG.K

a Spot No. is the unique number of the position where the spot display in the master gel;

b The number of peaks that match the trypsin peptides;

c The number of peaks that do not match the trypsin peptides;

d Protein score based on combined mass/mass spectrums;

e Each spot corresponding to a certain protein had at least one of the shown peptides identified.

### Gene Transcription Profile Analysis by Quantitative Real-Time PCR (qRT-PCR)

**Figure 3:**
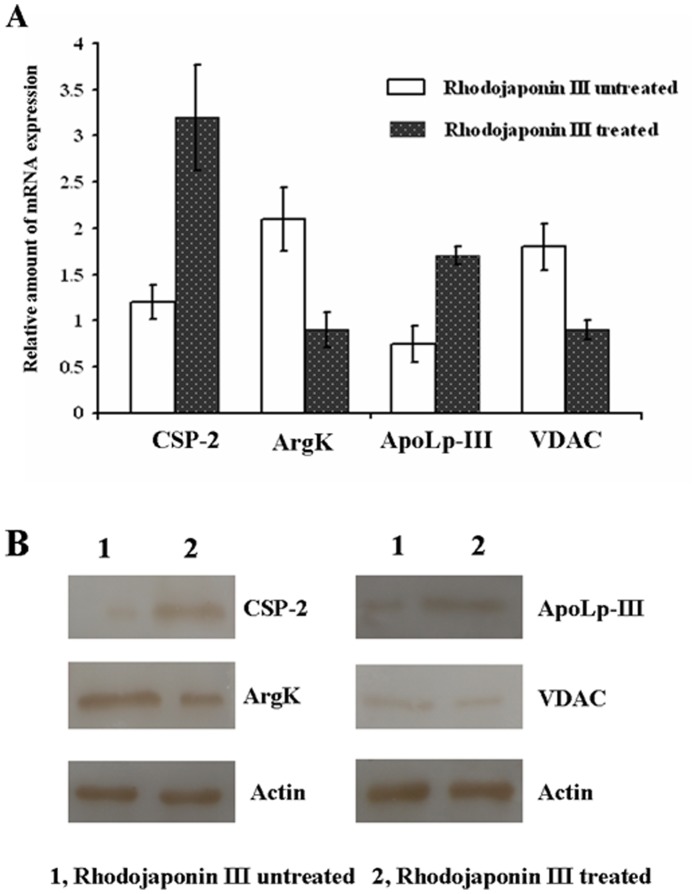
Validation of differentially expressed proteins in control and exposed to rhodojaponin III moths (PxCSP-2, PxArgK, PxApoLp-III and PxVDAC genes were selected randomly). A, qRT-PCR, B, Western blot. The mRNA level was normalized relative to the actin transcript. Each point represents the mean value ± S.E.M of three independent experiments with three individuals in each replicate.

To confirm the LC-MS/MS results, we used qRT-PCR to examine gene transcription. Four different genes from *P. xylostella* at 48 h after adult emergence were selected randomly for the analysis. These primer designs were based on the available sequences on NCBI GenBank. The gene-specific primers are listed in Table S1, and actin was chosen as an internal control. As shown in Figure 3, four genes showed consistent mRNA and protein expression patterns. The results suggested that these four genes expression patterns are consistent with the protein levels identified in proteome.

### Knockdown of PxCSP-2 Results in Disoriented Oviposition Behavior

In order to confirm the function of the CSP-2, RNAi analysis was used. After injecting synchronously 4^th^ larva groups with DEPC water, 0.5 µg dsGFP, 0.1 µg PxCSP-2dsRNA, or 0.5 µg PxCSP-2dsRNA, total survival rates after emergence were 86.67%, 84.44%, 82.22% and 77.78%, respectively. To investigate the efficiency of RNAi after ingestion of dsPxCSP-2 in *P. xylostella*, *PxCSP-2* mRNA levels were measured by qRT-qPCR in adults collected 1, 2, 3, 4 d after emergence. And the PxCSP-2 protein was investigated by Western blot in adults collected 2 d after emergence. The transcript levels of *P. xylostella* were decreased by 28.57–76.69% 1–4 d after injection of ds*PxCSP-2* RNA compared to the DEPC water and dsGFP treated group. The putative PxCSP-2 bands were considerably more intense in the DEPC water and dsGFP treated groups than in the 0.5 µg PxCSP-2dsRNA treated group, but the actin bands showed no change. This result confirmed that RNAi-mediated knockdown of *PxCSP-2* was highly effective (Fig. S1). And other CSPs expression had an increasing tendency while *PxCSP-2* expression reduced markedly post-injection (Fig. S2).

**Figure 4:**
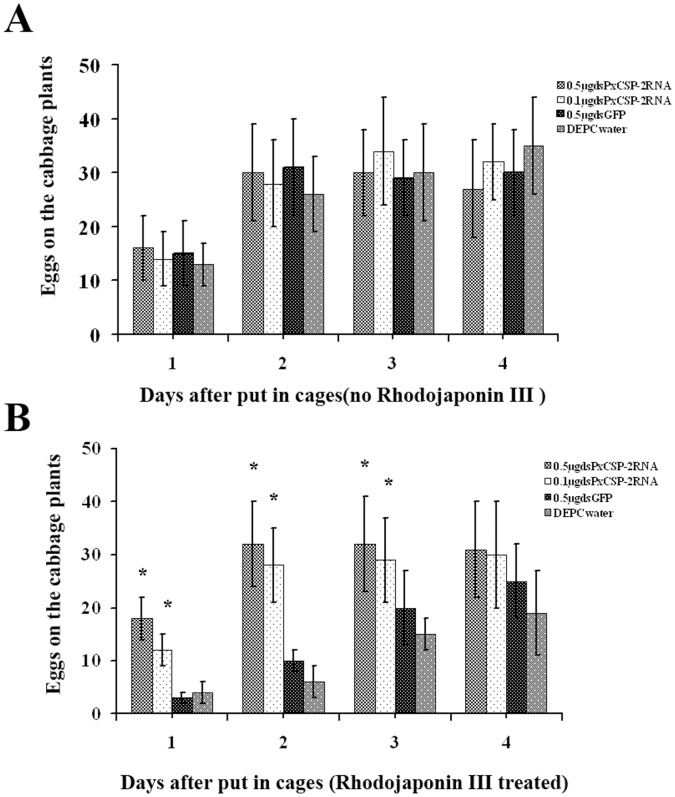
RNAi-mediated knockdown of *PxCSP-2* gene confused oviposition behavior (twenty-five pairs were analyzed per group). (A) Rhodojaponin III untreated; (B) Rhodojaponin III treated. The data represent the mean values ± S.E.M of three replicates. ‘*’ means statistically significant difference in number of eggs on the cabbage leaves compared to DEPC water (*t*-test, p<0.05).

After the insects emerged in 24 h, we successfully allocated them into 25 pairs per group. The number of eggs from every individual female adult was counted every day for four continuous days. The results demonstrated that PxCSP-2 plays an important role in the recognition of rhodojaponin III in *P. xylostella*. Moths treated with 0.5 µg of *PxCSP-2* dsRNA showed a 69.23% increase in oviposition compared to those injected with the DEPC water and 0.5 µg dsGFP (Fig. 4).

## Discussion

The use of rhodojaponin III as an insecticide is well documented. The rhodojaponin III was confirmed to possess feeding-deterring, oviposition-deterring and insecticidal properties [12], [13], [15], [26]. Sensitivity between species to the effects of rhodojaponin III is profound. Report had testified that exposure of *P. xylostella* at concentration 0.5 g/L of rhodojaponin III exhibited an intense oviposition-deterring activity [15]. This concentration of rhodojaponin III was concerned in this study. The oviposition step is crucial in the Lepidoptera insects because the hatching larvae are often relatively immobile and depend on the judicious choice of a host plants by the adult female [4]. It is particularly important for larvae survival, and the rhodojaponin III has ovicide activity [27]. Therefore, elucidating the mechanism of how the insect response to the rhodojaponin III is a promising approach to control the insect.

There are many binding proteins on these sensilla, such as odorant-binding proteins (OBPs) and chemosensory proteins (CSPs), and these proteins bind compounds in the environment [5], [6]. Cuticular hydrocarbons, plant volatiles and their derivatives are highly hydrophobic compounds and therefore cannot diffuse through the hydrophilic lymph to reach the dendritic membrane. OBPs in the sensillar lymphis were postulated to mediate the solubilization of hydrophobic odorant molecules, and thereby to facilitate their transport to the receptor neurons [17]. The OBPs expression in cells located in pheromone or general-odorant sensitive sensillae [28]. CSPs are ubiquitous soluble small proteins expression in sensory organs of many insect species, which are believed to be involved in chemical communication [29]. In insects, CSPs share no sequence homology with either PBPs or general OBPs [30], [31]. Although there were no direct evidences that they play an important role in olfactory or taste, their tissue location and initial ligand binding data both support the hypothesis that CSPs are involved in chemoreception. Their natural ligands have not yet been reported, although binding data indicate that CSPs bind highly hydrophobic linear molecules similar to insect pheromones and fatty acids [32], [33].

The oviposition step is crucial in the Lepidoptera insects because the hatching larvae are often relatively immobile and depend on the judicious choice of a food plant by the adult female, It is particularly important for larvae survival [4]. In the RNAi experiment, when the *PxCSP-2* message was silenced, the moths failed to discriminate insecticide rhodojaponin III on the cabbage plants and the oviposition behavior was confused. The oviposition increased 81.25% compared to the DEPC water injected group in the second day averagely (Fig. 4). In this study, PxCSP-2 protein was significantly increased after the moths exposed to rhodojaponin III, but the OBPs were not changed. We had reported that CSP protein in *S. litura* had significant affinity to rhodojaponin III with CDOCKER program and fluorescence emission spectra [34]. These results provided more evidences that CSP-2 might be the direct critical protein which binds some in-volatile hydrophobic plant secondary metabolites such as rhodojaponin III and guides the oviposition behaviour.

The odorant receptors (Ors) belong to the large superfamily of G protein-coupled receptors (GPCRs), which detect chemicals in their environment [35]. Interestingly, in the 2-DE analysis, odorant receptor proteins were no change, knockdown the Orco (formally known as OR83b) gene in the *S. litura* could result in disoriented oviposition behavior [36]. These results might support the idea that the Orco function is not required for some chemicals recognition per se, but it plays a role in context dependent recognition of them [37]. But, how the CSP-2 interacted with odorant receptors needed more understood.

In this study, we found a series of proteins changed in the moths exposed to rhodojaponin III beside energy metabolic and stress response proteins. The sensory neurons of the olfactory epithelium are among the few cells of the nervous system in direct contact with the external environment. Therefore, the olfactory neurons are exposed to both odorants and xenobiotics directly [38]. Given the importance of the olfactory system in controlling critical behavior patterns such as feeding and egg laying (oviposition), the olfactory systems had evolved both mechanisms for detoxifying xenobiotics to minimize cytotoxicity and mechanisms for inactivating odors to minimize signal saturation. Many odor degrading enzymes had been found in insect antennae, such as carboxylesterase, aldehyde oxidase, cytochrome P450 oxidoreductase, glutathione S-transferase, etc [38]–[41]. There are 3 glutathione S-transferase protein spots (spots 22, 23 and 26) were significantly altered when treated with rhodojaponin III compared to the untreated control. These results show that the glutathione S-transferase proteins might play an important role in degradating rhodojaponin III in the sensitive sensillae and avoiding the body damage.

Lipophorin is the main lipoprotein found in the hemolymph of insects, it transports phospholipid, diacylglycerol, and hydrocarbons among insect tissues [42]. Lipoproteins and molecules for pattern recognition are essential in the innate immune response of both vertebrates and invertebrates. The apolipophorin III (apoLp- III) is a major exchangeable lipid transport molecule found in the blood (hemolymph), may also play a crucial role in the innate immune response and act in pattern recognition response and multicellular encapsulation reactions [43]. Apolipophorin III can stimulate increase in hemolymph antibacterial activity and superoxide production by hemocytes [44], [45]. In *M. sexta*, the apoLp- III was dramatically up-regulated during the programmed death of insect skeletal muscle and neurons [46]. The 14-3-3 zeta protein was immune-stimulated in hemocytes by baculoviral infection in *Heliothis virescens*, but the mechanism is remain unknow [47]. Enolase is not only a key factor for controlling energy metabolism, but also is an immunosuppressive factor involved in phosphoenolpyruvate synthesis. In this study, apoLp- III, 14-3-3 zeta and enolase were significantly up-regulated after exposed to rhodojaponin III (spot 15, 21, 28). These data implicated that rhodojaponin III might stimulate the innate immune system of *P. xylostella*.

Arginine kinase-like protein (ArgK), which is less abundant after exposed to rhodojaponin III, is a phosphotransferase that catalyzes the reaction between L-arginine and ATP to produce L-phospho-arginine and ADP. It is the only phosphagen kinase in insects, which plays a vital role as ATP-buffering systems to regulate ATP level, just like creatine kinase in vertebrate [48]. The voltage-dependent anion-selective channel (VDAC) also was down-regulated, VDACs transport adenine nucleotides and other anions and metabolites across the outer mitochondrial membrane in eukaryotes, The ‘closed state’ remain permeable to small anions but are impermeable to organic ions such as ATP [49]. To enhance survival during unfavorable periods, in *H. armigera*, the ArgK activity was maintained at low level in diapausing pupae but rose in nondiapause pupae [50]. Other important energy metabolism-related enzyme expressions were changed too, like acyl-CoA dehydrogenase, glyceraldehyde-3-phosphate dehydrogenase et al. This implies that the insect may change energy metabolism to endure the harsh condition stress the after exposed to rhodojaponin III.

In this study, only the abundant proteins were monitored and many other less abundant proteins may also play a role involved in chemoreception and other function. And some unknown proteins my have important functions. Recently developed RNA deep sequencing technologies, such as Solexa/Illumina RNA-seq and Digital gene expression (DGE), might facilitate the investigation of the functional complexity of transcriptomes [51].

### Conclusions

Co-evolution between the insects and plant is a complex biological process rather than a simple gene expression change. The present results indicate that there is a potential interaction between rhodojaponin III and the insect defense responses. Our study contributes to the further understanding of potential molecular mechanism of co-evolution between insects and plants.

## Materials and Methods

### Ethics Statement

No specific permits were required for the described studies. No specific permissions were required for these locations. The location we collected the insects is not privately-owned or protected in any way. The insects used in the studies did not involve endangered or protected species. During the experiment, we never maltreated the insect.

### Insect Culture

The Insects were collected from insecticide-free cabbage and brought to the laboratory for rearing. Larvae were maintained on the cabbage leaves [*Brassica campestris* L.ssp. Chinensis (L·)] in a rearing room with conditions set at 25±1°C, 16∶ 8 h light : dark photoperiod and 70–80% relative humidity. The newly emerged adults were transferred to new cabbage plant for oviposition and added honey as a dietary supplement.

### Protein Samples Preparation and Two-Dimensional Gel Electrophoresis Analysis

For each sample group, 100 mg of adult *P. xylostella* moths (females) was ground into powder in liquid nitrogen. The powder was transferred to a clean eppendorf tube, adding 1 mL of a precooled lysis buffer (7 M urea, 2 M thiourea, 4% 3-[(3-cholamidopropyl)-dimethylammonio]-1-propane sulfonate (CHAPS), 30 mM Tris-HCl, and protease inhibitor cocktail), and homogenized on ice, then sonicated (10×15 s pulses) on ice. The homogenate was centrifuged centrifugation (12000 rpm) for 30 min at 4°C. Supernate was transferred to other clean eppendorf tube, The protein was precipitated with cold acetone at –20°C for 4 h, the protein precipitate was washed with cool pure acetone for two times (12,000 g, 15 min, 4°C), then was dried for about 5 min using vacuum drier and redissolved in rehydration buffer (8 M urea, 2 M thiourea, 4% CHAPS, 100 mM dithiothreitol (DTT), and 2% ampholyte). The protein concentrations were determined by the Bradford method (Bio-Rad, Hercules, CA). For 2-DE, 500 µL (1 mg) of protein was loaded was loaded in 17 cm, pH 3–10 IPG strips (Bio-Rad) for isoelectric focusing. The IEF program as follows: active rehydrate at 20°C, 50 V for 12 h, a linearly increasing gradient from 0 to 100 V for 1 h, speediness increasing to 200 V for 0.5 h, linearly increasing to 1000 V for 0.5 h, linearly increasing to 4000 V for 1.5 h, speediness keeping 4000 V for 6000 Vh, and electric current for each strip limited to 50 µA.

For SDS-PAGE, the gel strips were equilibrated for 15 min in equilibration continuously for 15 min with equilibration solution I (6 M urea, 0.375 M Tris-HCl, pH 8.8, 20% glycerol, 2% SDS and 20 mg/mL DTT) and then the equilibration solution II (25 mg/mL iodoacetamide instead of DTT). The equilibrated strips were run on 12% SDS-polyacrylamide gels at 10 mA per gel for 1 h and 50 mA per gel until the bromphenol blue (sealing the IPG gels with agarose sealing solution, containing 0.5% agarose, 0.1% SDS, 25 mM Tris-HCl, 0.001% bromophenol) front reached the bottom of the gel and electrophoresis was performed at 18°C. This experiment was performed for at least three times.

### Silver-stained, Image Analysis and MS/MS

After 2-DE, the gels were fixed in 10% (v/v) acetic acid and 30% ethanol (fixing solution) for 30 min, and then stained with AgNO_3_ solution [52]. The gel images were scanned using Umax scanner and analyzed quantitively with PD-quest version 8.0 analysis software (Bio-Rad, Hercules, CA). Protein spots displaying ≥1.5 average-fold increase or decrease in abundance (p-value <0.05) were selected for protein identification. The identified spots were excised from the gel and digested in gel as reported in Shevchenko et al [53]. Briefly, the gel particles were washed in de-ionized water twice (10 min each), placed in 100% CH_3_CN, and then dried in a speed vacuum. Dried gel pieces were covered with 10 µL of 12.5 ng/µL sequencing grade trypsin (Promega) in 25 mM NH_4_HCO_3_ buffer. In-gel digestion was incubated at 37°C overnight. Each 2.5-µL sample was spotted on an AnchorChip plate (Bruker Daltonics) followed by 1 µL of 0.4 mg/mL HCCA in 70% acetonitrile and 0.1% TFA. Samples were analyzed using Ultraflex III TOF/TOF mass spectrometer (Bruker Daltonics). External calibration was performed using Bruker peptide calibration standards. Mass spectra (MH+) were acquired by FlexControl (version 3.0, Bruker Daltonics) which recorded in the range 800–4,500 Da and the MS/MS information was obtained in LIFT (laser-induced forward transfer) mode.

### Database Searching

The identification of the proteins separated by 2-DE was performed on the World Wide Web (WWW). Peptide mass fingerprints (PMF) of the tryptic peptides from MALD-TOF/TOF MS/MS data on differential spots, together with the isoeletric points and molecular weights were combined by BioTools software (version 3.1, Bruker Daltonics). The data were searched against the EST database of *P. xyllostella,* NCBInr database and NCBI EST_others database (taxonomyof Metazoan) to obtain information, and results that were statistically significant (p<0.05) were accepted.

### RNA Extraction and cDNA Synthesis

Total RNA was isolated from twenty individual adults using Trizol reagent according to the manufacturer’s specifications (Invitrogen, USA). First-strand cDNA was synthesized with a first strand synthesis kit using Reverse transcriptase M-MLV (RNase H^–^) (TaKaRa, Japan). Briefly, 0.5 µg of total RNA, 1 µL of Oligo (dT) primer (50 µM), and the addition of RNase free deionized H_2_O was added up to 6 µL, 70°C 10 min and chilled with ice more then 2 min immediately. Then 0.5 µL of RTase M-MLV (RNase H^−^), 2 µL of 5×M-MLV buffer, 0.5 µL of dNTP Mixtrure (each 10 mM), 0.25 µL RNase Inhibitor and the addition of RNase free deionized H_2_O was added with the final volume 10 µL. The reaction protocol was performed at 42°C for 60 min, 70°C for 15 min, and cooled with ice. These cDNAs were stored at −20°C.

### Quantitative Real-time PCR (qRT-PCR)

The primers used for quantitative real-time PCR (qRT-PCR) are listed in Table S1. Aliquots (0.5 µL) of the synthesized first-strand cDNA were amplified by PCR in 20 µL reaction mixtures using an iCycler iQ (BIO-RAD, Hercules, CA) and SYBR Premix Ex Taq (Takara, Japan). The reaction conditions consisted of: 94°C for 2 min, followed by 40 cycles of 94°C for 5 s, 55°C for 10 s, and 72°C for 15 s. The actin gene (gi|117970201) was used as an internal standard. After the amplifications, a melting curve analysis was performed in triplicate and the results were averaged. The values were calculated using three independent biological samples, and the well-known 2^−ΔΔCT^ method was employed for the analysis of relative gene expression [54].

### Expressions of Recombinant and Polyclonal Antibodies Production

The four genes (PxCSP-2, PxApoLp-III, PxArgK and PxVCDA) cDNA fragment sequences were amplified with specific primer-pairs (Table S3), which contain the restriction sites *Bam*H I and *Hin*d III, respectively. The PCR product was excised with *Bam*H I and *Hin*d III and then subcloned into the pET28a(+) (stored in laboratory of insect toxicology, South China Agricultural University) vector. The recombinant proteins was expressed in BL21(DE3) competent cells induced by 0.6 mM IPTG. The *E. coli* pellet was solubilized in 6 M urea in 50 mM Tris-Cl buffer, pH 8.0 and then purified with a Ni-NTA column (GE Healthcare). Purified recombinant proteins were respectively used to immunize rabbits as described previously [55]. These sera of the immunized rabbits were collected as the polyclonal antibodies. These serum titers were detected by an enzyme linked immunosorbent assay (ELISA) [56]. And these antibodies had no cross-reactivity.

### Western Blot

A BCA kit was used for Western-blotting analysis, and the method was modified according to the methods previously described [57]. Briefly, a total of 300 µg of whole body proteins were separated on a 12% SDS-PAGE gel; the gel was semi-dry transferred for 40 min at 10 volts to an Immobilon-P PVDF membrane (Millipore, Bedford, USA). Immunoblotted with anti-PxCSP-2 serum (diluted 1∶1500), anti-PxApoLp-III serum (diluted 1∶1200), anti-PxArgK serum (diluted 1∶3000) and anti-PxVCDA serum (diluted 1∶1800), then an IgG goat anti-rabbit antibody conjugated with HRP was used for a secondary antibody (BOSTER, Wuhan, China, 1∶5000 dilution). Non-specific binding was blocked using a 5% fat-free milk solution.

### RNA Interference and Bioassay

To verify the specificity of RNAi for PxCSP-2 gene, the dsPxCSP-2 fragment (342 bp) was aligned with the other CSP proteins, and 19-bp consecutive identical sequences between them were not found. According to the manufacturer recommendations of T7 RiboMAX™ Express RNAi System (Promega), two pairs of primers (T7PxCSP-2F and PxCSP-2R, PxCSP-2F and T7PxCSP-2R) (Table S2) were designed to synthesize the 342-bp (61–402 bp) region of the *PxCSP-2* gene that included a T7 promoter region in both the sense and antisense strands. The *PxCSP-2* cDNAs from the whole moths were used as a template. The amplification reaction protocol comprised preheated 94°C for 4 min, then 36 cycles of 94°C for 35 s, 56°C for 40 s and 72°C for 60 s, with a final extension step of 72°C for 5 min. The sequence was verified by sequencing (Invitrogen Company, Shanghai, China). The *GFP* gene (ACY56286) was used as a control dsRNA. The PCR primers GFPF and GFPR were used to amplify the *GFP* fragment (688 bp), and dsRNA was synthesized by the T7 RiboMAX™ Express RNAi System. The final dsRNA product corresponding to the *PxCSP-2* gene (ds*PxCSP-2*) was eluted into DEPC water, stored at −80°C and used within 1 week.

The 4^th^ larvae were injected with 2 µl of construct containing 0.1 or 0.5 µg ds*Px*CSP-2RNA using a microINJECTOR™ System MINJ-1 (Tritech Research, Los Angeles, CA, USA). In addition, two controls were performed, an equivalent volume of dsGFP and DEPC water. In the target gene detection experiment, each group had 80 individuals with three replicates, and 10 moths were selected randomly at 1 d, 2 d, 3 d and 4 d after emergence for independent mRNA detection. An individual with more than a 10% decrease of the target gene expression was regarded as an effective RNAi, which was used to calculate the efficiency of RNAi. In the oviposition behavior analysis, each group had 25 pairs of moths with three replicates and were transferred to the cabbage plants, and the observation was performed every 24 hours for four continuous days.

### Rhodojaponin III Treatment and Bioassay

Rhodojaponin III (min. 95% AI) was extracted and purified from dried flowers of yellow azalea (*Rhododendron molle* G. Don) using silica gel [13] and stored in the laboratory of insect toxicology (South China Agricultural University). The purity was analyzed with HP1100 (USA), and the standard was obtained kindly from the Utah Natural Products Research Institute (NPI). To make the emulsifiable concentrate, 0.1 g extract was dissolved in 100 mL acetone with 2 g APSA-80. In the preliminary work, we did not find it [APSA-80] had a significant effect on the oviposition behavior. The preparation was dispersed in ultrapure water with a final concentration of 0.5 g/L rhodojaponin III, and the emulsions were applied evenly on the leaves and stalks of the cabbage with a small brush (there was no rhodojaponin III in the control group). After blow-drying the solvent, each cabbage was covered with two ends of an open transparent plastic cylinder. The cylinder was 50 cm in height and 25 cm in diameter, and the tops were enclosed with a piece of nylon mesh to prevent the insects from moving in or out. The newly emerged adults were collected, and each female was matched with one male in each pot of the cabbage. Each experiment utilized 25 pairs in three or replicates. The number of eggs on the whole was recorded everyday for continuous 4 days. For protein analysis, 48 h continuous exposed moths on treated cabbage plants were collected, and frozen in liquid nitrogen.

### Statistical Analysis

Statistical calculations were performed using SPSS software statistical software (Version 13.0; SPSS, Inc., USA). All data are expressed as the means (± S.E.M.). The significance of the difference in means was determined by two-tailed Student *t*-test to identify significant differences at a 95% confidence level (“*”, p<0.05).

## Supporting Information

Figure S1 **Detection of the efficiency of RNAi and the impact on** ***PxCSP-2*** **mRNA levels and protein levels by RT-qPCR (A) and Western blot (B), respectively.** (A) The relative expression levels of *p. xylostella* CSP-2 mRNA after different treatments. The data represent the mean values±S.E.M of three replicates. ‘*’ means statistically significant difference in expression levels compared to DEPC water (*t*-test, p<0.05). (B) Western Blot analysis. Immunoblotted with anti-CSP-2 serum (diluted 1∶1500) and visualized by ECL. Actin was used as an internal control. 1, DECP water; 2, dsGFPRNA; 3, 0.1 µg dsPxCSP-2RNA; 4, 0.5 µg dsPxCSP-2RNA.
(TIF)

Figure S2 **Detection of the relative expression levels of other CSPs in the** ***p. xylostella*** **after RNAi of** ***PxCSP-2*** **by RT-qPCR.** A, PxCSP-1, B, PxCSP-3, C, PxCSP-4, D, PxCSP-5. The data represent the mean values±S.E.M of three replicates. ‘*’ means statistically significant difference in expression levels compared to DEPC water (*t*-test, p<0.05).
(TIF)

Table S1 **Primers for Quantitative Real-Time PCR Measurements of Expression Levels of Selected Genes.**
(DOC)

Table S2 **Primers used in RNAi.**
(DOC)

Table S3 **Primers used for recombinant expressions.**
(DOC)
