# Supplementary figures and images for: Proteomic and Properties Analysis of Botanical Insecticide Rhodojaponin III-Induced Response of the Diamondback Moth, Plutella xyllostella (L.)
Source: PLoS One. 2013 Jul 5;8(7):e67723. doi: 10.1371/journal.pone.0067723 (PMC3702551; doi:10.1371/journal.pone.0067723)

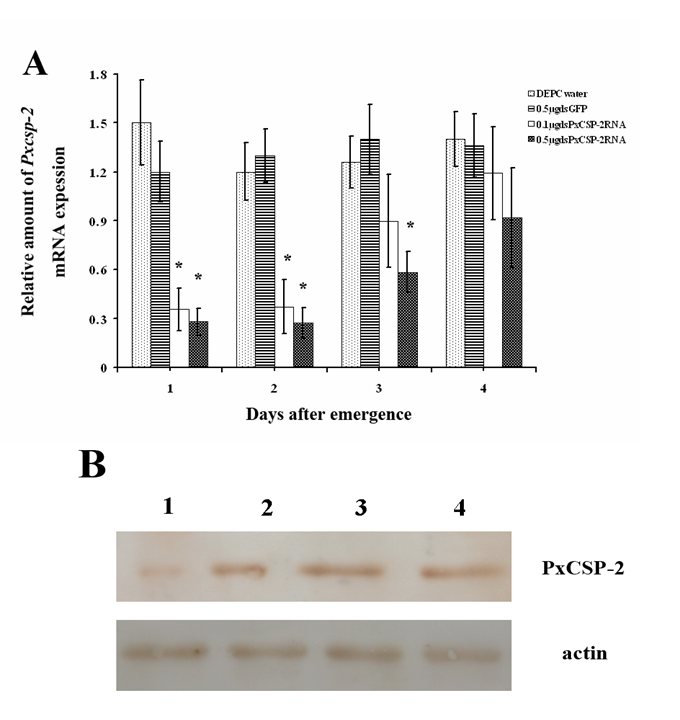

Supplement: Figure S1 — Detection of the efficiency of RNAi and the impact on PxCSP-2 mRNA levels and protein levels by RT-qPCR (A) and Western blot (B), respectively. (A) The relative expression levels of p. xylostella CSP-2 mRNA after different treatments. The data represent the mean values±S.E.M of three replicates. ‘*’ means statistically significant difference in expression levels compared to DEPC water (t-test, p<0.05). (B) Western Blot analysis. Immunoblotted with anti-CSP-2 serum (diluted 1∶1500) and visualized by ECL. Actin was used as an internal control. 1, DECP water; 2, dsGFPRNA; 3, 0.1 µg dsPxCSP-2RNA; 4, 0.5 µg dsPxCSP-2RNA. (TIF) [file pone.0067723.s001.tif]

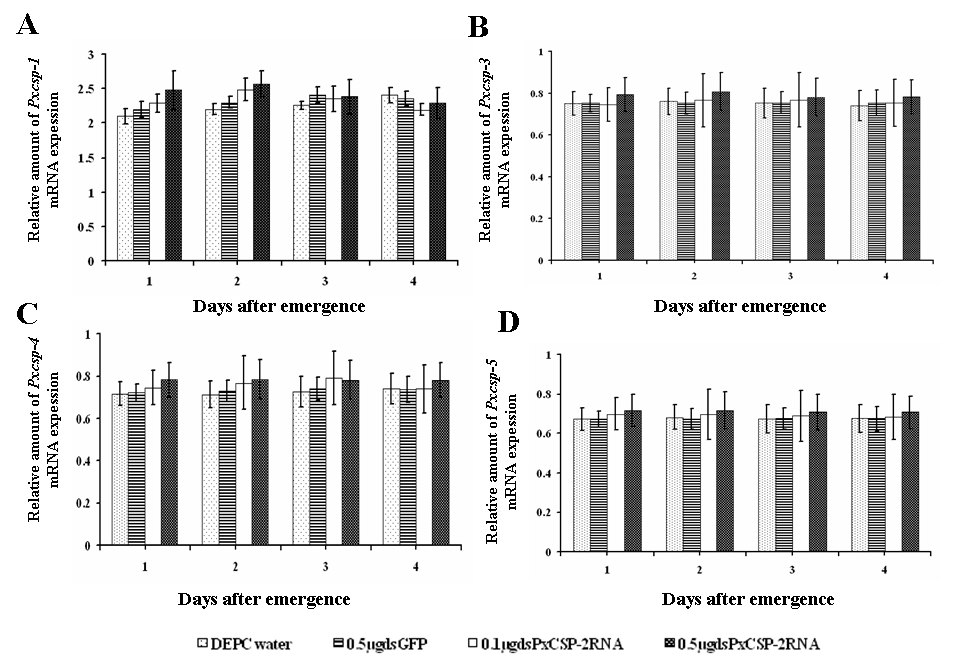

Supplement: Figure S2 — Detection of the relative expression levels of other CSPs in the p. xylostella after RNAi of PxCSP-2 by RT-qPCR. A, PxCSP-1, B, PxCSP-3, C, PxCSP-4, D, PxCSP-5. The data represent the mean values±S.E.M of three replicates. ‘*’ means statistically significant difference in expression levels compared to DEPC water (t-test, p<0.05). (TIF) [file pone.0067723.s002.tif]
