# Supplementary material for: Proteomic and Properties Analysis of Botanical Insecticide Rhodojaponin III-Induced Response of the Diamondback Moth, Plutella xyllostella (L.)
Source: PLoS One. 2013 Jul 5;8(7):e67723. doi: 10.1371/journal.pone.0067723 (PMC3702551; doi:10.1371/journal.pone.0067723)
Supplement: Table S1 — Primers for Quantitative Real-Time PCR Measurements of Expression Levels of Selected Genes. (DOC) [file pone.0067723.s003.doc]

Table S1. Primers for Quantitative Real-Time PCR Measurements of Expression Levels of Selected Genes

| accession No. | gene name | primer (5’-3’) | product length |
| --- | --- | --- | --- |
| gi|209978476 | CSP-2 | sense: AAGCTAACCCTGGCTTGTCTC | 198 |
|  |  | antisense: GATGTGCTCCTTCAGCTCCTT |  |
| [gi|301508512](http://www.ncbi.nlm.nih.gov/protein/301508512?report=genbank&log$=prottop&blast_rank=1&RID=SFR9G03501N) | apoLp-III | sense: TTCCAGAAGGAGTTCTCCAAGC | 181 |
|  |  | antisense: TCTTCTCCAGCGCCTCCTTC |  |
| gi|284927832 | ArgK | sense: GAAGACTTCCTTCGGCTCCA | 209 |
|  |  | antisense: TTCCGAGGGTCTCAACATCAC |  |
| KC477227 | VCDA | sense: GTCCAAATACGCCGTCAAAG | 200 |
|  |  | antisense: TGAAGGCAGCCAAGTCAGTC |  |
| GI:122894080 | CSP-1 | sense: TTGATAGGGAAGGCAGTATGTGA | 175 |
|  |  | antisense: CGTCCGGCAGAGTATGTTTTAG |  |
| GI:124246513 | CSP-3 | sense: TAGTATGCCTTGCCCTGGTG | 192 |
|  |  | antisense: AGCCTCTTTGATGTGAGATTTGA |  |
| GI:124246515 | CSP-4 | sense: CGCCAAGTACGACAGCTTCA | 197 |
|  |  | antisense: TCACCACGACTCGCACCAG |  |
| GI:124246516 | CSP-5 | CGACTCCCATTACACCGACA | 207 |
|  |  | ACCTTGCGTGTACCATTCTTTT |  |
| gi|117970201 | Actin  (internal control) | sense: CCATCTACGAAGGTTACGCTCTT | 192 |
|  |  | antisense: CCATCTCCTGCTCGAAGTCC |  |
