# Supplementary material for: Proteomic and Properties Analysis of Botanical Insecticide Rhodojaponin III-Induced Response of the Diamondback Moth, Plutella xyllostella (L.)
Source: PLoS One. 2013 Jul 5;8(7):e67723. doi: 10.1371/journal.pone.0067723 (PMC3702551; doi:10.1371/journal.pone.0067723)
Supplement: Table S2 — Primers used in RNAi. (DOC) [file pone.0067723.s004.doc]

Table S2. Primers used in RNAi

| For *Px*CSP dsRNA synthesis | |
| --- | --- |
| *Px*CSP-2F | 5’-AACGACTCCCACTACACCGACC-3’ |
| *Px*CSP-2R | 5’-CACCAGCCAGCAACATGAGG-3’ |
| T7*Px*CSP-2F | 5’-ggatcctaatacgactcactatagggAACGACTCCCACTACACCGACC -3’ |
| T7*Px*CSP-2R | 5’- ggatcctaatacgactcactatagggCACCAGCCAGCAACATGAGG -3’ |
| For GFP dsRNA synthesis | |
| GFPF | 5’-AAGGGCGAGGAGCTGTTCACCG-3’ |
| GFPR | 5’-CAGCAGGACCATGTGATCGCGC-3’ |
| T7GFPF | 5’-ggatcctaatacgactcactatagggAAGGGCGAGGAGCTGTTCACCG-3’ |
| T7GFPR | 5’-ggatcctaatacgactcactatagggCAGCAGGACCATGTGATCGCGC-3’ |
