# Supplementary material for: Proteomic and Properties Analysis of Botanical Insecticide Rhodojaponin III-Induced Response of the Diamondback Moth, Plutella xyllostella (L.)
Source: PLoS One. 2013 Jul 5;8(7):e67723. doi: 10.1371/journal.pone.0067723 (PMC3702551; doi:10.1371/journal.pone.0067723)
Supplement: Table S3 — Primers used for recombinant expressions. (DOC) [file pone.0067723.s005.doc]

Table S2. Primers used for recombinant expressions

| For *Px*CSP recombinant expression | |
| --- | --- |
| *Px*CSPEF | CA*GGATCC*ATGCAGAAGCTAACCCTGGCT |
| *Px*CSPER | GA*GGATCC*TTACACCAGCCAGCAACATGA |
| For *Px*ArgK recombinant expression | |
| *Px*ArgKEF | CA*GGATCC*ATGGTGGACGCTGCAACTCTT |
| *Px*ArgKER | GA*GGATCC*TTACAGGGACTTCTCGATCTTG |
| For *Px*ApoLp recombinant expression | |
| *Px*ApEF | CA*GGATCC*CAGCTGCAGGACCTGGAGAA |
| *Px*ApER | GA*GGATCC*TCACTGTTTGCTGGCGGCGT |
| For *Px*VDAC recombinant expression | |
| *Px*VDACEF | CA*GGATCC*CTCCTCCATTCTACGCTGA |
| *Px*VDACER | GA*GGATCC*GGGCTCCAGCTCCAGGGC |
